# Using text mining techniques to extract phenotypic information from the PhenoCHF corpus

**DOI:** 10.1186/1472-6947-15-S2-S3

**Published:** 2015-06-15

**Authors:** Noha Alnazzawi, Paul Thompson, Riza Batista-Navarro, Sophia Ananiadou

**Affiliations:** 1 National Centre for Text Mining, Manchester Institute of Biotechnology, 131 Princess St., Manchester, M1 7DN, UK; 2 Jubail University College, Royal Commission for Jubail and Yanbu, Jubail City, Saudi Arabia, 10074; 3 Department of Computer Science, University of the Philippines Diliman, Quezon City, Philippines, 1101

## Abstract

**Background:**

Phenotypic information locked away in unstructured narrative text presents significant barriers to information accessibility, both for clinical practitioners and for computerised applications used for clinical research purposes. Text mining (TM) techniques have previously been applied successfully to extract different types of information from text in the biomedical domain. They have the potential to be extended to allow the extraction of information relating to phenotypes from free text.

**Methods:**

To stimulate the development of TM systems that are able to extract phenotypic information from text, we have created a new corpus (PhenoCHF) that is annotated by domain experts with several types of phenotypic information relating to congestive heart failure. To ensure that systems developed using the corpus are robust to multiple text types, it integrates text from heterogeneous sources, i.e., electronic health records (EHRs) and scientific articles from the literature. We have developed several different phenotype extraction methods to demonstrate the utility of the corpus, and tested these methods on a further corpus, i.e., ShARe/CLEF 2013.

**Results:**

Evaluation of our automated methods showed that PhenoCHF can facilitate the training of reliable phenotype extraction systems, which are robust to variations in text type. These results have been reinforced by evaluating our trained systems on the ShARe/CLEF corpus, which contains clinical records of various types. Like other studies within the biomedical domain, we found that solutions based on conditional random fields produced the best results, when coupled with a rich feature set.

**Conclusions:**

PhenoCHF is the first annotated corpus aimed at encoding detailed phenotypic information. The unique heterogeneous composition of the corpus has been shown to be advantageous in the training of systems that can accurately extract phenotypic information from a range of different text types. Although the scope of our annotation is currently limited to a single disease, the promising results achieved can stimulate further work into the extraction of phenotypic information for other diseases. The PhenoCHF annotation guidelines and annotations are publicly available at https://code.google.com/p/phenochf-corpus.

## Background

Phenotypes constitute the visible properties of an organism that are produced by the interaction of the genotype and the environment (i.e., skin colour and height) [1]. A greater understanding of phenotype-disease associations is needed to determine the etiology of such diseases, which can enhance disease prevention and treatments [2].

The study of disease-phenotype relationships has been hampered by the scarcity of suitable large-scale, machine-readable knowledge bases. Existing resources, such as the Online Mendelian Inheritance in Man (OMIM) [3] and the Human Phenotype Ontology (HPO) [4] are manually constructed, making them difficult to update and maintain. They could, however, be enriched by exploiting the vast amounts of phenotypic information available in various textual sources, including the ever-growing volumes of published biomedical literature, and patient EHRs, which have proliferated with the consolidation of modern healthcare systems [5]. EHRs contain both structured/coded and unstructured information pertaining to patient morbidity, treatment and care over time [6], which can enhance understanding of disease etiology and phenotype-genotype associations [7]. On the one hand, coded data (e.g., unique identifiers from medical controlled vocabularies [8]) lends itself well to computational processing, but comes with limited expressivity, possibly resulting in loss of vital details regarding patient conditions. On the other hand, unstructured data (e.g., progress notes, discharge summaries and radiology reports), written as narrative text, provides greater detail about patient conditions, such as diagnoses, findings, signs and symptoms, procedure, family history, etc. [9]. The increasing volume of unstructured text, however, presents challenges, both to clinical practitioners and to computerised applications used for clinical research [7], since the phenotypic information "locked" within the text must be located and correctly interpreted. There is thus an urgent need to develop TM methods that can automate the extraction and integration of vital phenotypic information hidden in narrative text, to help to derive information about disease correlations and thus support clinical decisions [10].

Developing TM tools for use in new domains is reliant upon textual corpora, in which pertinent information has been explicitly marked up by experts. Such annotated corpora serve both as training data for machine learning (ML) techniques [11,12] and as a gold standard for systematic evaluation of new methodologies.

Whilst TM techniques have been widely applied in the extraction of relationships involving genes and proteins from the biomedical literature [13,14], there has been little research into the extraction of disease-phenotype relationships, either from the literature or from EHRs. This is largely due to the lack of suitably annotated EHR corpora, owing both to their sensitive data and the difficulty of applying de-identification techniques. However, a small number of publicly available, de-identified clinical corpora have been released recently (e.g., [15-17]).

To stimulate research into the automatic extraction of phenotypic information from text, we have developed a new corpus (PhenoCHF), annotated with various types of information relating to phenotype-disease associations by two medical doctors. To our knowledge, the corpus is unique, both in the detail of the phenotypic information annotated, and in that it integrates different text types, i.e., literature articles and discharge summaries from EHRs. The high quality of the annotations is illustrated by inter-annotator agreement levels of up to 0.92 F-score.

The inclusion of heterogeneous text types in PhenoCHF aims to encourage the development of robust TM systems that can extract comprehensive phenotypic information from multiple sources with differing characteristics. For example, EHRs exhibit non-standard grammatical structure and high levels of lexical and semantic variability, coupled with many domain-specific abbreviations, complex sentences [18], as well as spelling errors (around 10% of words) [19].

PhenoCHF focusses on a specific medical condition, i.e., congestive heart failure (CHF), which occurs when the heart does not supply all body parts with a sufficient amount of blood to carry out their usual functions. This focus is motivated by CHF's current standing as the world's most deadly disease [4] and it being a comorbidity of renal failure [6]. Through annotation of phenotypic information surrounding CHF, we aim to facilitate the development of TM-based systems that can highlight the role of CHF in kidney function deterioration, identify the risk factors for CHF and find patients with lower or higher risk factors.

In this paper, we extend upon our previously reported work [20], which was focussed only on the construction of PhenoCHF. Specifically, using an augmented version of the corpus, we have carried out experiments to train systems to recognise phenotypic information automatically, employing different ML algorithms and feature sets. Encouraging results have been achieved through evaluation on both the PhenoCHF corpus and the partially overlapping ShARe/CLEF 2013 corpus.

In the remainder of this paper, we firstly provide an overview of related work and highlight the novel aspects of our work. Subsequently, we provide a detailed description of the research methods employed in constructing and annotating PhenoCHF, and in the application of different TM techniques to PhenoCHF to facilitate automatic phenotype extraction. Finally, we discuss and compare the results of the different TM methods employed.

### Related work

Over the last decade, significant advances in biomedical TM have resulted in a shift in research focus from the recognition of entities to the extraction of more complex information from biomedical literature, e.g., interactions between proteins [2] and more detailed relationships between drugs, genes and cells [21,22]. The initial focus on abstracts has been recently extended to include full literature articles containing much more detailed information. Although the BioNLP Shared Tasks [23] in particular have encouraged the development of sophisticated machine learned TM systems through the release of annotated corpora covering different biomedical subdomains, these do not address disease-phenotype associations.

Due to the lack of publicly available clinical corpora, most existing TM systems operating on clinical texts (e.g., [23,25]) employ dictionary-based methods, aiming to map mentions of clinical concepts within the texts to entries in the UMLS Metathesaurus [26]. Such techniques can match concept mentions whose lexical forms are related to entries in UMLS, but they cannot detect their *semantic *variants. A further issue is that the UMLS Metathesaurus does not include semantic categories corresponding directly to phenotypic information. Although the phenotype-specific HPO appears more suitable in this respect, it only covers a subset of human diseases.

As an alternative to purely dictionary-based approaches, rule-based methods [3] involve developing regular expressions which can combine dictionary-based information with orthographic and lexical characteristics of targeted entities and their surrounding contexts. Although their expressive power provides greater coverage than dictionaries, manual rule formulation can be time consuming, and the resulting rule set is likely to be over-tuned to the development corpus.

ML methods, such as hidden Markov models (HMMs) [27], maximum entropy Markov models (MEMMs) [28] and conditional random fields (CRFs) [29], can be *trained *to recognise entities, also using a range of textual characteristics (e.g., orthography [30], parts-of-speech (POS) [21], affixes [22] and dictionary-based information). These approaches can learn implicit patterns in annotated data, allowing them to draw better generalisations than manually constructed rules. CRF models in particular have been demonstrated to exhibit superior performance in several information extraction tasks dealing with both biomedical literature (e.g., [31]) and clinical text [15], the latter in the context of the Integrating Biology and the Bedside (i2b2) 2010 concept extraction task, which involved the automatic recognition of treatments, problems and tests [15]. The i2b2 corpus constitutes one of the few publicly available, semantically annotated clinical corpora. A further related corpus was released as part of the ShARe/CLEF 2013 NER task [32].

Only a small amount of previous work has focussed specifically on the extraction of phenotypic information from narrative text. The MedLEE system [25], originally aimed at extracting information from clinical text, was adapted to extract phenotypic information from ambulatory care notes [33] and biomedical literature [34]. In another study, a set of 100 molecular biology articles was annotated with phenotypic information in a semi-supervised manner, and then used to evaluate a hybrid phenotype extraction approach based on dictionary and ML techniques [35].

Our research differs from the studies above in a number of ways. Firstly, previous related work has tended to focus on extracting all instances of phenotypic phenomena regardless of context, e.g., diseases, signs and symptoms or anatomical parts. In contrast, our work concentrates on a specific disease, i.e. CHF, but aims to extract detailed phenotypic information surrounding this disease. Secondly, whilst other efforts have focussed either on clinical texts or biomedical literature, our study is the first that integrates the results of applying TM to both text types. Thirdly, we have produced the first expert-annotated corpus with detailed phenotypic information to stimulate a shift from dictionary-based approaches to ML-based ones. As new terms are introduced frequently, manually curated domain-specific dictionaries cannot be relied upon to identify all relevant concepts that occur within text.

To demonstrate the utility of our PhenoCHF corpus in training ML-based phenotype extraction systems, we present a comparative evaluation of different NER methods, i.e., rule-based, dictionary-based and ML approaches. We show that machine learned models exhibit competitive performance when compared to rule-based methods, especially when different text types are used as training data. The portability and robustness of our best performing machine learned model is tested through its evaluation on another corpus (i.e., the ShARe/CLEF 2013 data set, which we were allowed to use after having completed the NIH training course), whose annotation scope partially overlaps with PhenoCHF, but which includes a wider range of free-text reports from EHRs including discharge summaries, electrocardiogram, echocardiogram and radiology reports.

## Methods

The first part of this section describes the creation of the PhenoCHF corpus, including the document selection process, the design of the annotation scheme, the production of the guidelines and evaluation of the quality of the expert-produced annotations. We then provide a detailed description of the different named entity recognition (NER) methods we have implemented to extract phenotypic information from PhenoCHF.

### Selection of documents for PhenoCHF

The major part of the PhenoCHF corpus consists of 300 discharge summaries of patients who are known to suffer from CHF as a major complaint, and also from kidney failure. These discharge summaries constitute the subset of the 889 de-identified discharge summaries released as part of the i2b2 recognising obesity challenge [17] (to which we obtained access by signing a data use agreement) that contain mentions of our target diseases, either in their full forms, as acronyms (e.g., *CRF, CRI*) or as synonyms (e.g., *renal insufficiency, kidney failure*). The second part of the corpus consists of the 10 most recent (at corpus collection time) full-text articles retrieved from the PubMed Central Open Access database, using the query we have provided in Additional File 1.

### PhenoCHF annotation scheme and guidelines

To capture various types of phenotypic information relating to CHF (Table 1), we have designed a multi-level annotation scheme that identifies entities relevant to phenotypic phenomena, as well as important relationships that hold between these entities. The involvement of a cardiologist in the design has helped to ensure the relevance of the scheme to our research goals.

**Table 1:** Annotated phenotype concept types.

Entity Type	Description
Cause	Any medical problem that contributes to the occurrence of CHF
Risk factors	A condition that increases the chance of a patient having the CHF disease
Sign & symptom	Any observable manifestation of a disease which is experienced by a patient and reported to the physician
Non-traditional risk factor	Conditions associated with abnormalities in kidney functions that put the patient at higher risk of developing "signs & symptoms" and causes of CHF

Every textual mention of a concept relevant to the description of phenotypic information relating to CHF is annotated at two different levels. Firstly, an appropriate semantic category is assigned by the annotators. Secondly, the manually identified terms are mapped semi-automatically to known clinical concepts in the UMLS Metathesaurus, with the aid of MetaMap [24]. As a final level of annotation, several types of relationships that hold between the concept mentions are manually annotated. Annotators were supported by concise guidelines, developed in an iterative manner, together with regular meetings to allow the discussion of issues that arose during the annotation process. Acting as adjudicator in these meetings, the cardiologist was responsible for resolving all problems and discrepancies.

**Figure 1:**
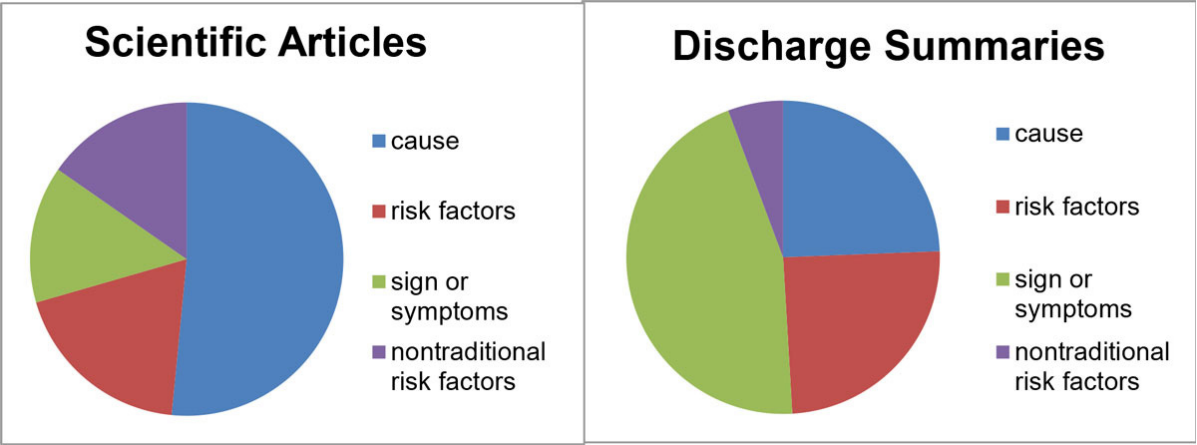
**Distribution of phenotypic information types in the corpus**. Phenotypic concepts were manually annotated by our domain experts.

Figure 1 shows the most prevalent phenotypes in the corpus and their distribution in the discharge summaries and articles. In discharge summaries, there is large emphasis on describing the signs and symptoms of the disease, but these play a much less significant role in scientific articles, where the dominant topics are non-traditional risk factors and the etiology of CHF.

### Reliability of annotations

The employment of two different annotators allowed us to calculate the inter-annotator agreement (IAA) rate, to verify the quality of the annotations. Since the widely used Cohen's kappa [36] is not suitable in this case because the total number of annotated items is not known in advance, we took one set of annotations as the gold standard and calculated F-score values to measure IAA.

Table 2 reports the IAA rates achieved in PhenoCHF, differentiating between agreement levels according to text type, and also between agreement rates for *exact matching *(i.e., the boundaries of annotated terms must match exactly) and *relaxed matching *(i.e., the boundaries of annotated terms need only overlap with each other). The F-scores for exact matching are generally lower than for relaxed matching, due to disagreements between annotators with regard to the exact span annotated, e.g. whether or not modifiers should be included within annotated text spans. For example, whilst one annotator marked up *significant left atrial dilation *as an expression corresponding to sign or symptoms of CHF, the other annotator marked up only *left atrial dilation*.

**Table 2:** Inter-annotator agreement on the PhenoCHF annotations.

	Discharge summaries		Articles	
**Phenotypic class**	**Exact**	**Relaxed**	**Exact**	**Relaxed**
Cause	0.84	0.95	0.59	0.78
Risk factor	0.84	0.94	0.86	0.79
Sign & symptom	0.69	0.97	0.53	0.82
Non-traditional risk factor	0.77	0.83	0.81	0.72
Macro-average	0.82	0.92	0.69	0.77

F-scores were calculated using both exact and relaxed matching.

### Phenotype extraction

The annotated PhenoCHF corpus was subsequently used in experiments to extract and categorise mentions of concepts relating to phenotypes automatically. The recognition of such concept mentions is a prerequisite for the extraction of more complex information (e.g., relations involving these concepts). Whilst PhenoCHF is also annotated with such relations, their automatic extraction is left as future work.

Extracting phenotype concept mentions from text is a typical NER task, which involves determining the boundaries of the mentions, and assigning semantic types to them. We have developed and evaluated different NER methods, i.e., dictionary-based, rule-based and ML algorithms. To support this, each part of the corpus was divided randomly into a training set (80%) and test set (20%). Due to the small size of the corpus, we did not set aside a development or validation set. However, to address this shortcoming, we also validated our ML-based methods using cross validation.

The dictionary-based method, our baseline, involved applying MetaMap to PhenoCHF. Although, as mentioned above, the dictionary underlying this software (i.e., UMLS Metathesurus) does not have any explicit phenotypic semantic categories, we know that phenotypic information usually falls under the subtypes of the Metathesaurus' *disorder *semantic group, confirmed both by previous studies (e.g., [35]), as well as by our annotators. Since, by default, MetaMap annotates a broad range of clinical concepts, we configured it to recognise only those concepts belonging to the 12 categories under the disorder semantic group, e.g., *disease or syndrome *and *pathologic function*, in order to match only phenotypes.

For the rule-based approach, we exploited Conceptual Annotations for Facts, Events, Terms, Individual Entities and Relations (Cafetiere), a system that allows the application of rules to free text [37]. The system provides a web-based interface that allows rules to be defined and applied to texts. Further development of rules is facilitated in Cafetiere through the highlighting of rule-added annotations within texts, and an indication of the identifier of the rule that was used to create each annotation.

Through manual examination of examples of different phenotypic phenomena and their contexts in the training portions of PhenoCHF, we used Cafetiere to develop a set of hand-crafted rules (45 and 37 rules for discharge summaries and articles, respectively), which use a range of features including syntactic (i.e., POS), semantic (UMLS semantic type) and lexical (word shape, prefix and suffix) to capture common textual patterns that denote the presence of phenotype information. The performance of the rules was evaluated through their application to the test portions of the PhenoCHF corpus. A sample rule is shown in Example 1, which will recognise phrases such as *heart is enlarged , abdomen was distended*, and *leg is swollen*.

Example 1.

[syn=NN|NNP]{1,3},

[sem=beverb]?,

[syn=VBN|JJ, token!="normal"|"regular"|"stable"]

Each rule consists of a sequence of token specifications to be matched in the text. Each token specification is enclosed within square brackets. In Example 1, the first type of token to be matched should be syntactically either a common noun (NN) or proper noun (NNP). The iterator {1,3} allows the rule to match a sequence of nouns, to capture signs and symptoms which are expressed using compound nouns (e.g., *chest pain, swollen leg*). The second, optional token (as indicated by the question mark) to be matched is any form of the verb *be*. Finally, a verb in past participle form or an adjective must be present. The optionality of *be *accounts for different possible writing styles (i.e., *leg swollen *or *leg was swollen*).

Our ML approach used three different algorithms, i.e., HMMs, MEMMs and CRFs. The annotated data was first transformed into the begin-inside-outside (BIO) format. We then employed CRFsuite [38], the Mallet implementation of MEMM [39] and, since the traditional HMM does not assume independent features [40] (hence each observation is independent from its context), we adapted a previous HMM-based approach [41] to allow for the integration of multiple features. To perform a fair comparison between the three algorithms, we used the same set of features to train the models, including word level information (e.g., bag-of-words), syntactic information (e.g., POS and chunk tags obtained using the biomedical model of the state-of-the-art GENIA tagger [42], which has been shown to achieve precision of 97-98% for POS tagging on biomedical text), and word affixes, i.e., prefixes and suffixes. The affix-capturing features were introduced based on the observation that certain prefixes and suffixes are quite common amongst phenotypic expressions, e.g., the prefix *hyper-* in *hypertension *and the suffix *-emia *in *lipidemia *and *anemia*. Using the gold standard annotations in the training set, a registry of prefixes/suffixes of lengths two to five was first automatically compiled (following [43]), serving as a look-up list during feature extraction. In this set of experiments, we trained various models using different feature sets in conjunction with each ML algorithm on the training part of the corpus, and evaluated the models on our test set, using bag-of-word features as the baseline. Separate sets of experiments were carried out for the different text types in the corpus, i.e., discharge summaries and literature articles.

A subsequent set of experiments aimed to determine the extent to which a model trained on one text type is robust to the features of alternative text types. We trained a model on the set of PhenoCHF discharge summaries and tested it on all of the scientific articles, and vice versa. Furthermore, in order to report results which aim to eliminate the possibility of models overfitting the training data, we performed five-fold cross validation on PhenoCHF's records and articles combined.

The portability of the models trained on PhenoCHF was furthermore demonstrated through their application to the corpus released for the NER task of ShARe/CLEF 2013 [32], whose annotations partially overlap with those in PhenoCHF. In the former, mentions of disorder terms were annotated and mapped to corresponding concepts in the SNOMED CT terminology [44], whose disorder semantic group covers phenotypic information. However, the annotated information in ShARe/CLEF corpus is much broader in subject scope than PhenoCHF, whose annotations (and hence, systems trained using the corpus) are restricted to information concerned with heart disease. Thus, to provide a fair evaluation of our models against the ShARe/CLEF corpus, we applied them only to a subset (i.e., 135 and 76 records from the training and test sets, respectively) which contains terms mapped to concepts within the "heart disease" subtree of SNOMED CT.

## Results and Discussion

Table 3 shows the results of the various NER methods, i.e., dictionary-based (MetaMap), rule-based (Rules) and the three machine learning algorithms (MEMM, HMM and CRF), when applied separately to the different text types in the corpus (i.e., discharge summaries and literature articles).

**Table 3:** Comparison of different methods developed and evaluated on the PhenoCHF training and test sets, respectively.

	Discharge summaries						Articles					
	**Exact Match**			**Relaxed Match**			**Exact Match**			**Relaxed Match**		

**Methods**	**P**	**R**	**F**	**P**	**R**	**F**	**P**	**R**	**F**	**P**	**R**	**F**
Dictionary (MetaMap)	0.22	0.29	0.25	0.39	0.51	0.44	0.42	0.25	0.30	0.67	0.33	0.44
Rules	0.88	0.86	**0.87**	0.92	0.93	**0.92**	0.83	0.88	**0.85**	0.88	0.90	**0.89**
MEMMs	0.67	0.33	0.52	0.87	0.60	0.54	0.18	0.55	0.24	0.20	0.56	0.28
HMMs	0.90	0.63	0.74	0.90	0.65	0.76	0.30	0.55	0.39	0.32	0.58	0.41
CRFs	0.88	0.77	0.82	0.90	0.86	0.88	0.48	0.62	0.54	0.53	0.69	0.60

For MEMMs, HMMs and CRFs, only the results from the model with the best performing combination of features are presented (P=precision, R=recall, F=F-score).

Rules achieved the highest F-score for both parts of the corpus, and outperformed the other methods, as shown in Table 3. However, it is important to note that this method is also the most costly, since the manual examination of textual patterns that is required to construct the rules can be very time-consuming.

Most of the false negatives resulting from the rules can be attributed to phenotype information that is not present in the training data, and hence was not accounted for by the rules. The false positives, meanwhile, are due to some non-phenotype terms sharing similar syntactic patterns to phenotypic terms, which the rules failed to discriminate. For instance, the rule in Example 1 will incorrectly recognise the phrase *abdomen is benign *because many signs or symptoms are expressed using the same syntactic pattern, e.g., *abdomen is distended*. Although the rule tries to filter out phrases that refer to normal conditions by excluding certain patterns in the training data (e.g., *chest is normal, heart is regular*), it failed to filter out the unseen phrases matching the rule in the test data.

Meanwhile, the dictionary-based method produced a greater number of false positives, even though we restricted the semantic types recognised by MetaMap to those belonging to the disorder group. Whilst MetaMap recognised all disorders, such disorders were only annotated in PhenoCHF if they were mentioned in the context of CHF. Furthermore, MetaMap suffers from low recall due to some spelling mistakes in the corpus (i.e., *aneamia *instead of *anaemia*) and multi-word phenotypic expressions that MetaMap segments into different terms (e.g., *worsened renal function *split into *worsened *and *renal function*). Recall is even worse using exact matching, mainly because, unlike systems trained using PhenoCHF, MetaMap was not designed to recognise phenotypic information expressed as multi-word expressions with modifiers, e.g., *moderate to mild cough*.

Results obtained by the ML-based methods are very competitive with those from the application of our rule-based method, especially on the discharge summaries. As CRFs achieved the best performance on both the discharge summary and article subsets of PhenoCHF, we used only this method to train models in further experiments.

Whilst CRFs achieved the highest F-scores, MEMMs produced the lowest scores, having suffered from its known label bias problem [40], in which the model develops a bias towards classes with fewer outgoing transitions, thus requiring a very large amount of training data. Overall, our experiments demonstrate that ML methods exhibit good levels of precision, but suboptimal recall. This is partly due to the fact that they are sensitive to textual heterogeneity, such as the use of different vocabulary, e.g., synonyms and term variants, and different writing styles [40].

Regarding the contribution of different features to the performance of the ML models (provided as Additional File 2), we have observed that the POS and chunk features contributed little to improving the overall performance of the system, whilst the highest performance was achieved when prefix and suffix features were incorporated. A possible reason is that distinctive sets of prefixes and suffixes typically occur within terms referring to phenotypic information, e.g., the suffix -*emia *is especially common in risk factors (e.g., *hypercholesterolemia, hyperlipidaemia*).

For literature articles, the performance margin of the rule-based methods over ML is considerably greater than for clinical records. This can be explained by the smaller size of the article subset, and the greater scarcity of its annotations (compared to the clinical records corpus), which meant that ML models trained on this corpus subset had less observations to learn from.

Reinforcing this finding is our set of experiments which demonstrated that the model trained on the articles and tested on the discharge summaries performs with significantly lower F-score than the model trained on the discharge summaries and tested on the literature articles, as shown in Table 4. The specific and complex characteristics of EHRs mean that the literature-trained model exhibits even lower performance when applied to the different text type. In contrast, the larger size of the discharge summary portion of PhenoCHF, and the richer annotations contained within it, allowed for a more accurate model to be trained.

**Table 4:** Results of CRF model training and evaluation.

Evaluation data	Training Data	P	R	F
PhenoCHF Articles	Discharge summaries	0.79	0.47	0.58
PhenoCHF Discharge summaries	Articles	0.56	0.29	0.38
PhenoCHF (full) 5-fold cross validation		0.89	0.83	0.86

Experiments were performed using different document types (P=precision, R=recall, F=F-score)

This does not mean, however, that the literature part of the corpus is not useful for machine learning, since our best results are achieved by training and evaluating models using 5-fold cross validation on the pooled corpus of both records and articles, as shown in Table 4. This result is in contrast to other related studies (e.g., [18]) which have found that pooling corpora of different text types normally decreases the performance of the trained model. However, our results show that the annotation of heterogeneous textual sources according to a common set of guidelines can allow training of a single classifier that is robust to different text types.

The robustness of the model on different text types, together with the superiority of CRF models in this context, are further reinforced by the results obtained when applying the PhenoCHF-trained models to the ShARe/CLEF corpus. Again, the best results were obtained with the CRF model trained on the complete, pooled PhenoCHF corpus, as shown in Table 5. Most of the false positives found by our model are due to the discrepancies in the annotations contained in the two different corpora.

**Table 5:** Results from the application of PhenoCHF models on ShARe/CLEF.

	Training set			Test set		
**Method**	**P**	**R**	**F**	**P**	**R**	**F**
Record model	0.25	0.49	0.33	0.06	0.18	0.09
Article model	0.29	0.22	0.25	0.06	0.07	0.06
PhenoCHF model	0.25	0.53	0.34	0.07	0.18	0.10

Experiments were performed using our various CRF models (P=precision, R=recall, F=F-score)

To evaluate the extent to which the discrepancies between the output of our system and the annotations in ShARe/CLEF corpus are due to the different annotation scopes, our expert annotators reviewed both the false positives (FPs) and false negatives (FNs) output by our system, in comparison to the ShARe/CLEF annotations. The annotators identified how many of the FPs output by our system actually represent valid phenotypic information, and how many of the FNs represent information that is out of the scope of CHF (and hence could not be expected to be recognised by our system). This validation revealed that the majority of FPs recognised by our system represent valid phenotypic information in the context of CHF, and correspond to the wider range of semantic types that are annotated in PhenoCHF. In particular, our *signs and symptoms *category encapsulates the UMLS *finding *semantic type (e.g., *chest pain*), which is excluded from the ShARe/CLEF corpus. A smaller number of FPs was found to correspond to genuine errors made by our system. However, these were found to correspond largely to cases where non-phenotype terms share the same morphological form as correct phenotype terms. As an example, the suffix -*uria *is common amongst phenotypic information related to CHF, especially non-traditional risk factors (e.g., *dysuria*), but can also be used in non-phenotypic terms i.e., *cystnuria*. The FNs (e.g., *endocarditis*) were mainly due to the broader scope of ShARe/CLEF annotation, compared to the very focussed scope of PhenoCHF. A further source of error concerns acronyms and abbreviations. Although there are many examples in PhenoCHF, such as *CAD *(coronary artery disease) and *MR *(mitral regurgitation), there are also many abbreviations and acronyms in ShARe/CLEF that do not appear in PhenoCHF, e.g., *PAFIB, LBBB, CHB, PDA*. The high frequency with which these appear in the ShARe/CLEF test data set helps to account for the significantly lower F-score achieved by our models when applied to this data set.

**Figure 2:**
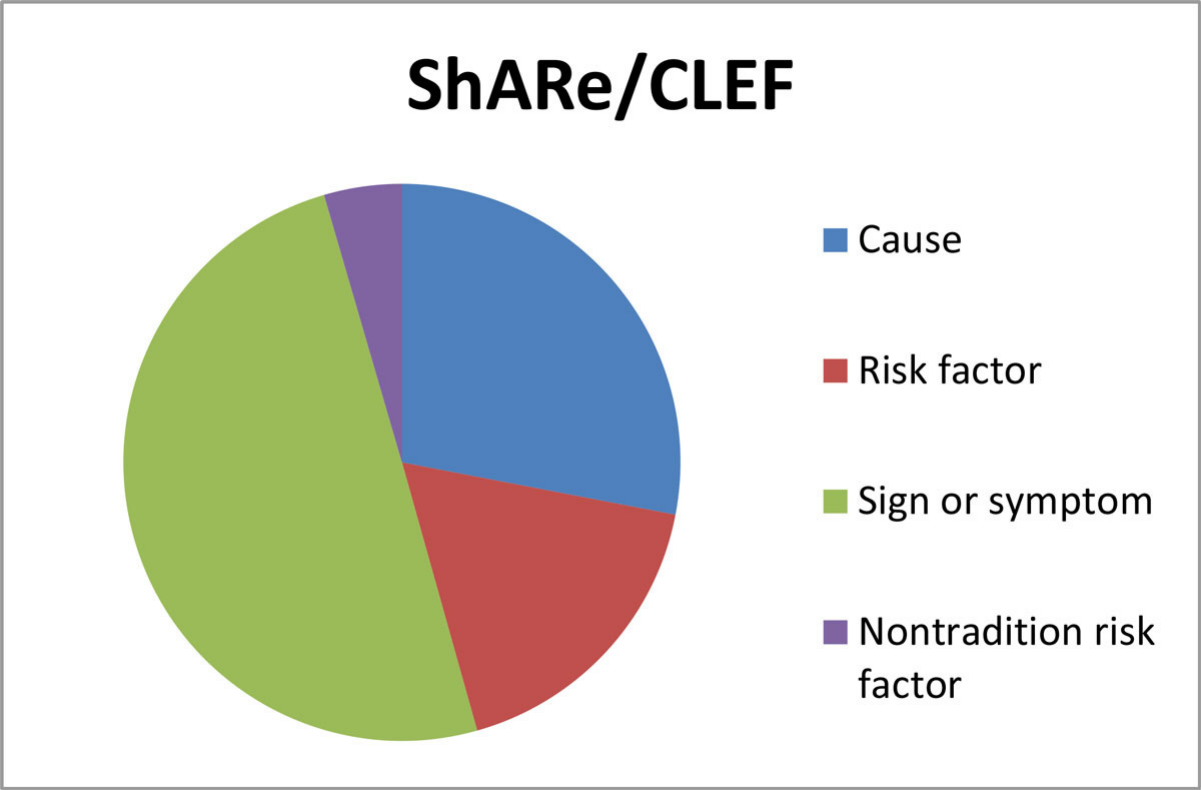
**Distribution of phenotypic information in the ShARE/CLEF corpus**. Phenotypic concepts were automatically recognised by our model and then validated by our domain experts.

When we remove the FPs that correspond to real phenotypic information, and the FNs that are out of the scope of our task, the revised precision and recall are 0.56 and 0.54, respectively and the F-score is improved to 0.55 for the training part of ShARe/CLEF, whereas the new precision and recall for the test part are 0.12 and 0.19, respectively, and the F-score is improved to 0.13. This provides evidence of the portability of our trained model to different domain-specific corpora, even when there are differences in text types between the corpora. The distribution of the types of phenotypic concepts relating to CHF in the ShARe/CLEF corpus, recognised by our model and validated by our experts, is shown in Figure 2. It is worth noting that the most prevalent phenotypic type is *sign or symptoms *followed by *cause*, whilst the least prevalent type is *non-traditional risk factor*.

## Conclusions

In this article, we have described our work towards facilitating the development of robust TM systems that can extract information relating to phenotypes from a range of different text types. We have developed the PhenoCHF corpus, which is annotated with information relevant to the identification of disease-phenotype associations in the context of CHF, including several types of entities and relationships that hold between them. The corpus aims to support the development of new, ML-based methods to identify phenotypic information in unstructured texts. Due to rapid advances in biomedicine, dictionary-based methods cannot support the recognition of new concept variants appearing in text. Machine learning methods, on the other hand, can recognise new concepts and variants, based on comparison of their textual and contextual features with known concept instances.

PhenoCHF includes literature articles and discharge summaries from EHRs, which have been annotated according to a common set of guidelines, to support the development of systems that can identify relevant phenotypic information from free text, regardless of the source or style of writing. To demonstrate the utility of PhenoCHF, we have shown that machine-learning NERs trained on the corpus can behave with superior performance to the more traditional dictionary-based methods. Whilst our experiments show that rule-based methods produce the best results, our best performing machine-learned classifier (CRF) achieves competitive performance, and alleviates the need for manual rule construction.

In terms of robustness, we have demonstrated that a system trained to recognise phenotype information in EHR records is able to achieve good levels of performance when applied to the same extraction task in literature articles. Further improvements in the portability of the system to multiple text types were achieved through training on a combination of literature articles and discharge summaries. This has been reinforced by the encouraging results achieved when applying our best-performing CRF model to a related, but wider-ranging corpus, i.e., the ShARe/CLEF corpus.

As future work, we will investigate the automatic extraction of relationships between entities, exploiting the relationship annotation that has been added by our expert annotators. We intend to employ the EventMine event extraction system [14], which has achieved superior performance compared to other state-of-the-art systems in several extraction tasks on biomedical literature [45]. Its adaptability to multiple text types and domains has been demonstrated, whilst recent improvements aim to simplify its configuration to new tasks, without the need for extensive additional coding [46].

## Competing interests

The authors declare that they have no competing interests.

## Authors' contributions

NA led and supervised the annotation, and implemented and evaluated the methods for phenotype extraction. PT contributed towards designing the annotation scheme and guidelines. RB participated in the design and implementation of the evaluation schemes. NA, PT and RB drafted the manuscript. SA supervised all steps of the work.

## Supplementary Material

Additional file 1 **Query used to retrieve the literature articles from the PubMed Central OpenAccess subset**. Click here for file

Additional file 2 **Table showing the contribution of features in each machine-learning based method**. Click here for file

## Declarations

The publication charges for this article were funded by the AHRC ("Mining the History of Medicine", Grant AH/L00982X/1) and the MRC ("Supporting Evidence-based Public Health Interventions using Text Mining", MR/L01078X/1).

This article has been published as part of *BMC Medical Informatics and Decision Making *Volume 15 Supplement 2, 2015: Proceedings of Louhi 2014: The Fifth International Workshop on Health Text Mining and Information Analysis. The full contents of the supplement are available online at http://www.biomedcentral.com/bmcmedinformdecismak/supplements/15/S2.
